# Recent Advances in Noninvasive Biosensors for Forensics, Biometrics, and Cybersecurity

**DOI:** 10.3390/s20215974

**Published:** 2020-10-22

**Authors:** Leif K. McGoldrick, Jan Halámek

**Affiliations:** 1 Department of Chemistry, University at Albany, State University of New York, 1400 Washington Ave, Albany, NY 12222, USA; lmcgoldrick@albany.edu; 2 Department of Environmental Toxicology (ENTX), Texas Tech University, 1207 Gilbert Drive, Lubbock, TX 79416, USA

**Keywords:** biosensors, forensics, biometrics, cybersecurity, fingerprints, sweat, blood, cipher

## Abstract

Recently, biosensors have been used in an increasing number of different fields and disciplines due to their wide applicability, reproducibility, and selectivity. Three large disciplines in which this has become relevant has been the forensic, biometric, and cybersecurity fields. The call for novel noninvasive biosensors for these three applications has been a focus of research in these fields. Recent advances in these three areas has relied on the use of biosensors based on primarily colorimetric assays based on bioaffinity interactions utilizing enzymatic assays. In forensics, the use of different bodily fluids for metabolite analysis provides an alternative to the use of DNA to avoid the backlog that is currently the main issue with DNA analysis by providing worthwhile information about the originator. In biometrics, the use of sweat-based systems for user authentication has been developed as a proof-of-concept design utilizing the levels of different metabolites found in sweat. Lastly, biosensor assays have been developed as a proof-of-concept for combination with cybersecurity, primarily cryptography, for the encryption and protection of data and messages.

## 1. Background

Biosensors are widely used in multiple processes today. These include, but are not limited to, clinical diagnostics [1,2,3,4,5,6,7,8,9,10], environmental processes [11,12,13], the food industry [13,14,15,16,17,18], and devices for military use [15,19,20]. More recently, the use of biosensors has been noted in other disciplines, namely forensics, biometrics, and cybersecurity. As biosensors are devices that employ sensing techniques relying on biorecognition elements, they are able to provide specific, rapid results pertaining to bioaffinity-based reactions. The use of biosensors in forensics enables investigators to have another source of information in addition to DNA analysis that also provides worthwhile information quickly for them to narrow down their investigation in a timely manner. Biometrics and biosensors are becoming more closely related as the technology improves in that field. The differentiation of people with more noninvasive biosensors, biosensors that do not involve intrusive procedures, is exceedingly useful. The main procedures used here involve electrochemical and enzymatic assays for analysis. Lastly, with the advent of computers, the use of biosensors in unconventional computing [21] and the combination of computing with chemistry, biology, and physics have become another facet for biosensors.

*Forensics*

In the realm of forensic science, there is an increasing need for new technologies to aid investigators and lab scientists in the pursuit of gathering worthwhile information from evidence. There are many subsections of evidence that are pertinent in the forensic field, but recent research has focused on three: Fingerprints, blood samples, and sweat-based field testing for ethanol and other drugs.

In the history of forensics, fingerprints have been essential in addition to being a widely emblematic feature of the forensic field in pop culture. However, fingerprints are mainly used in the field as a comparative means of identification [22], and if a print cannot be utilized for this pictorial comparison based on ridge structure, size, and shape, it is treated as exclusionary evidence [23]. This can be viewed as a large limitation on the amount of data that one can gain from this relevant piece of evidence. By analyzing the content of a print, namely the amino acids, one can gain some understanding of who the donor of that print was and be able to narrow down the search for the investigators. This is due to the metabolic [24] differences [25,26,27,28] in people due to their gender, age, medications, and lifestyle. By analyzing these types of biomarkers in a fingerprint sample, it would not only allow for one to gain much needed information that would provide additional context for investigators, but would also lead to the reduction in the need to wait for the lengthy analysis of DNA that causes a backlog [29], if any was recovered. According to the NIJ, a backlog is defined as any evidence that was not analyzed for at least 30 days after submission to a laboratory. By analyzing the content of a fingerprint instead of the pictorial fingerprint commonly relied on, it allows for smudged or partial prints, the prints that would not provide ample evidence for comparison, to have value for investigators. The chemical content of fingerprints has been examined as well, mainly focused on laboratory-based equipment such as mass spectrometric (MS) techniques that focus on total fingerprint content [24,30,31], drugs of abuse [32,33], and fatty acids to differentiate individuals based on age [34]. In addition to MS, there were optical techniques, as well using spectrophotometric instrumentation with age differences based on lipids [35], visual representation [36], and explosive content found on prints [37], as well as combined techniques such as desorption electrospray ionization (DESI) and direct analysis in real-time mass spectrometry (DART-MS) for total content [38,39,40] and for pictorial [41,42,43]. Further analysis of these techniques can be found in a review in Trends in Analytical Chemistry [44].

In addition to fingerprints, blood is another matrix that is commonly obtained as evidence for forensic investigators. In addition to the commonly known DNA matching with a database [45,46], bloodstains are also used for splatter analysis [47,48,49], and there are even techniques to differentiate if there are multiple overlapping stains [50]. An important quality relating to the bloodstain that was missing was the time since it was deposited onto the surface, utilizing a technique that is practical and did not require great sample prep or laboratory instrumentation. This is a vital piece of information as it would allow for corroboration of stories told by possible witnesses and would enable the reduction in irrelevant and unnecessary lab work to be done with blood that is too fresh or too old at a particular scene. Another lapse in analysis of bloodstains is the determination of the age of the person the blood is from, as this can be done with DNA but is a lengthy process [51]. Similar studies using biosensors were to find biological sex and ethnicity [52,53]. Other lab techniques were attempted to be able to perform age deduction; however, they were not applicable, due to serious flaws in the techniques [54].

The third forensic matrix is sweat. Sweat is a viable forensic sample for multiple reasons, as it contains a small amount of DNA [55,56,57,58], and other metabolites and compounds [59,60,61], and people leave traces of it upon contact of surfaces with their skin [62]. As fingerprints contain sweat in addition to other components, it is a comparable matrix to what has been discussed previously. Sweat can be detected in the field utilizing one of the main components in sweat—lactate [63,64,65,66,67]. Lactate is prominent in sweat samples and, even though there are techniques that can be used in order to detect it [68,69,70,71], they require complex laboratory instrumentation and are not viable methodologies for on-site deployment. Some of these techniques rely on a tattoo-like sensing device that consists of a potentiometric sensor with a wireless receiver [70,71], involving the use of a screen-printed electrode on tattoo paper with a microfluidic channel for sweat collection and detection of analytes. By being able to find sweat on surfaces directly at a crime scene, investigators would have additional samples of viable evidence. Building upon this, emerging research has also looked at analysis involving ethanol. Multiple studies have shown that ethanol is excreted into sweat [72,73] and that sweat cannot be tampered with, similarly to how people “trick” breathalyzers. There are wearable technologies that allow for biosensing of metabolites in sweat [74,75]; however, they have a long delay, some up to two hours, that makes them useless for on-site Driving While Intoxicated (DWI) analysis. Similar research has looked into saliva for the identification of tetrahydrocannabinol (THC) [76] and an overall approach that analyzes multiple types of legal and illegal compounds in sweat [77].

*Biometrics*

The second main discipline has been the use of biosensors for strictly biometric purposes. Namely, current research has aimed to use bioassays for the identification and differentiation of individuals. As mentioned previously, the content of different metabolites in sweat can be quantified, which can be used to find differences between people. This has implications in both the prior forensics section, biometrics in general, and even cybersecurity, which will be explained below. This method can be used to provide an alternate to DNA testing similar to the other techniques being developed for forensics. This kind of technology is similar to emerging research in biosensors and bioelectronics [78,79]. As mentioned previously, the content within a person’s sweat is a result of metabolic processes [24,25,26,27,59,80] related to what can be a person’s identifying factors such as age, biological sex, diet, and activity level. By taking measurements of and comparing results for multiple biomarkers, one can differentiate a person from others with these fluctuating factors. This can be applied for the unlocking of smart devices as the technology is moving in this direction as well [81,82,83].

As mentioned previously, sweat has been an emerging source of information in both forensics and biometrics. Many studies have been done for the advancement of various methodologies for different compounds found in sweat [84,85,86,87,88]. Further descriptions of some of the recent methodologies that have been developed for sweat analysis can be seen in Table 1 below.

*Cybersecurity*

The third and final discipline has been the use of biosensors for cybersecurity purposes. This is a small transition from biometrics into cybersecurity as they are closely related. This research can be applied in two different areas of cybersecurity—authentication and cryptography. Both are important in our world with the advent of the digital age, so there is a call for innovative and worthwhile technologies across all disciplines to innovate and advance the novel research into cyber technologies. For authentication, biometrics that were mentioned previously can be applied. Ideally, if one can differentiate people, the same technology can be used to identify a person, or at the very least, dismiss an imposter. Cryptography is the use of codes and cyphers in order to encrypt data to keep them safe, either in transmission between people as a message or safekeeping in storage [106,107,108]. Many multidisciplinary researchers have been applying their research to encryption, including, but not limited to, fluorescence [109,110,111,112,113,114,115,116,117], nuclear magnetic resonance (NMR) [118], bacteria [119], antibodies [120], and molecular computing systems [121,122,123,124], with the heaviest research in DNA applications [125,126,127,128,129,130,131,132,133].

## 2. Research

*Forensics*

**Figure 1:**
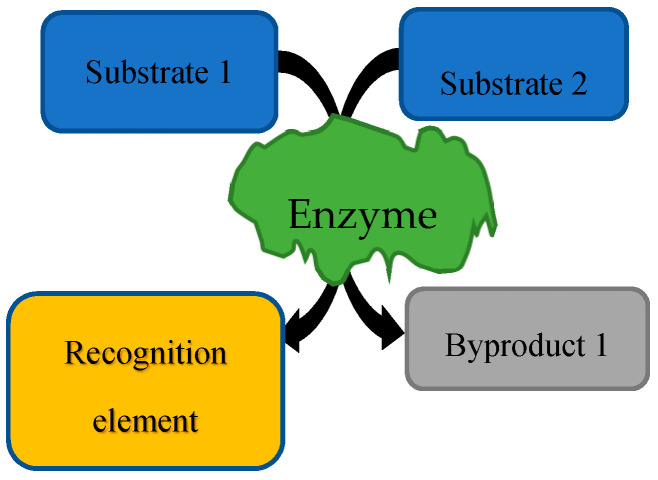
Generic enzymatic assay example.

First, in the forensic field, emerging research has focused on the three areas of study above with fingerprints [44,134,135,136,137], blood [138,139], sweat [140,141], and one general review on the use of biocomputing in forensics [142]. These five fingerprint papers provide novel applications of biorecognition elements that can be used for future biosensing devices. These papers are centralized on the idea that people have different levels of certain L-amino acids, which are related to their metabolism and different traits, allowing them to be differentiated into groups. The blood papers use the degradation of certain enzymes in order to determine the time that a blood spot has been outside the body and to identify the age of the originator from the level of a separate enzyme. The sweat papers use the levels of different compounds in sweat in order to identify sweat and to be able to provide an alternative technique for alcohol intoxication. The methodologies within these papers use different biosensors, some via enzyme assays, some with chemical reactions. A generic enzymatic assay diagram can be seen in Figure 1, where specific substrates for the enzyme are used in the assay to produce byproducts, one of which is a recognition element. The last paper is a review on other trending types of fingerprint analysis such as the use of mass spectrometry, spectroscopy, nanotechnology, and combinatorial methods [44].

A fingerprint paper from 2015 uses an enzymatic cascade utilizing L-amino acid oxidase (L-AAO) and horseradish peroxidase (HRP) in the presence of L-amino acids to produce a visible color by oxidizing the redox dye o-dianisidine, which results in a color that may be analyzed at 436 nm [134]. Due to the fact that women produce a higher amount of these amino acids than men [26,27,28], this assay would allow for the determination of a person’s biological sex. First, the assay was performed utilizing 50 mimicked samples, 25 male and 25 female, containing 23 target amino acids in concentrations based on reported values calculated in R-project software. This resulted in the area under the curve (AUC) of a receiver operating characteristic (ROC) curve of 99%, showing a high probability for this methodology to differentiate between the two sexes. This was then repeated with three male and three female volunteers testing both their right and left thumbs in an acid extraction methodology using polyurethane film as a medium that was developed in the same article. These real samples also show definitive differentiation between the male and female prints. Different surfaces around the lab, such as a computer screen and doorknob, were also tested in this manuscript to show viability on different surfaces. An additional paper from 2016 follows the same principles as the L-AAO/HRP assay in order to find an alternative methodology to determine biological sex, this time utilizing a chemical assay [135]. This assay uses ninhydrin, which produces a colorimetric reaction with alpha-amino (α-NH_2_) acids [143,144]. Ninhydrin is commonly used in forensics already as it produces Ruhemann’s purple as a product in the presence of all α-NH_2_ acids. The process in this paper is similar to the last: 50 total mimicked samples with designated concentrations of the 23 amino acids from R-project software are analyzed photometrically at 570 nm. The area under the ROC curve in this case was at 94%, showing a high probability to correctly distinguish the two sexes. After this, five male and five female volunteers were analyzed using this technique, resulting in a 91% area under the curve for authentic samples.

Following this trend, a paper from 2017 also uses a chemical assay for the determination of biological sex with the focus being on the Bradford reagent, Coomassie Brilliant Blue G-250 dye [136]. Bradford is commonly used for quantifying proteins and is less affected by reagents and nonprotein components of samples than other commonly used reagents [145]. The goal of utilizing this assay is that it only targets six specific amino acids with which to form a colorimetric complex, thus enabling a more focused approach to the determination of biological sex with the ideal being the determination of a single amino acid assay to differentiate the sexes. Contrary to the previous papers, only authentic fingerprints were sampled from 50 authentic fingerprint samples from volunteers—25 males and 25 females. This resulted in an area under the curve of the ROC graph of 99%, showing the highest probability of the three methods to correctly identify the biological sex of the fingerprint donor.

The final fingerprint paper to be examined goes one step further, by using two separate tests, each targeting a specific amino acid, in order to differentiate on the basis of biological sex [137]. The two methods used are an enzymatic cascade targeting alanine and a chemical assay that targets arginine. The alanine-targeting assay consists of a three-enzyme cascade with alanine transaminase, pyruvate oxidase, and horseradish peroxidase [146]. In the presence of alanine in addition to the other substrates necessary for the assay, a redox dye is oxidized by HRP and can be spectrophotometrically measured. Following the previously established standard, even though the mimicked samples produced the lowest AUC of the ROC curve with a value of 82%, the authentic samples of 50 total individuals was vastly improved at 99.8%. The second technique in this research consisted of the application of the Sakaguchi Test [147], which involves α-naphthol, NaOH, and sodium hypobromite in order to form a red-colored complex. As in the previous experiments, both mimicked and authentic samples were tested, resulting in both AUCs being 100%.

In order to apply these types of biosensor techniques to other bodily fluids that are forensically relevant, blood is another focus of research. The main topic that research on blood is centered around is the estimation of the time since deposition, TSD, of a blood spot. The first paper focuses on this by measuring the levels of two biomarkers in blood, citrate kinase (CK) and alanine transaminase (ALT), which denature with the passage of time of up to 5 days [138]. The CK assay involves creatine and adenosine triphosphate as substrates for the first enzyme, CK, in a three-enzyme cascade utilizing pyruvate kinase (PK) and lactate dehydrogenase (LDH) as the other two enzymes. LDH, the third enzyme in the cascade, is the enzyme involved with production of the recognition element β-nicotinamide adenine dinucleotide from β-nicotinamide adenine dinucleotide reduced, which produces a reduction in signal at 340 nm. ALT is a two-enzyme assay that also utilizes LDH to allow for simultaneous determination. ALT recognizes the substrates alanine and α-ketoglutaric acid. By using a two-analyte system, this provides a more reliable system of determination as it has parallel markers being analyzed compared to a single marker. Building upon this, the technique outlined in the second paper uses one assay to determine not only the TSD, but also an estimation of the age of the source individual [139]. By looking at alkaline phosphatase (ALP), this research achieves both goals. This is due to the fact that ALP is a commonly used biomarker in clinical diagnostics for bone growth that relates directly to the age of the individual. To measure this, ALP converts the substrate *p*-nitrophenol phosphate into *p*-nitrophenol, which also acts as the recognition element as *p*-nitrophenol is observable at 405 nm. As ALP is a biomarker for bone growth, and that it degenerates over time when out of the body, one can obtain data about the relative age of the blood donor and the TSD up to 2 days. For the analysis of this methodology, 100 samples were prepared via the R-project software mentioned previously, split evenly between young and old, males and females. The samples provided an AUC for the ROC curve of 99% for males and 100% for the female group in differentiating between old and young.

The third forensic medium that is being researched currently is sweat. As previously mentioned, sweat has many forensic applications but is difficult to identify at a crime scene. To this end, a novel methodology in order to identify sweat by use of a biosensor strip based on the detection of lactate was developed [140]. This method utilized an enzymatic assay in order to detect lactate, a major component in sweat. The assay used involved a two-enzyme cascade of lactate oxidase (LOx) and horseradish peroxidase (HRP). LOx involves the substrates lactate and oxygen, which are used to produce hydrogen peroxide. This hydrogen peroxide is then used by HRP as mentioned previously with a redox dye to produce a signal. This methodology was able to detect sweat with minimal decay for up to two weeks and at low amounts of sweat: Around 50 nL. This technique was even applied onto a paper strip modified with polystyrene for use as a field-deployable device. This optical strip provides a binary YES/NO for the presence of sweat via a color change, which is ideal for preliminary detection of sweat that can itself be analyzed further. Additionally, sweat was further examined in a noninvasive testing methodology for ethanol sensing on the surface of one’s skin [141]. This conceptualizes an alternative method to breathalyzers by relying on an enzymatic assay involving alcohol oxidase (AOx) and HRP that is quantified not only by UV-Vis spectrophotometry but also an optical camera. AOx uses ethanol and oxygen as substrates to produce hydrogen peroxide, which is used by HRP as mentioned previously. This research shows that there is a correlation between both techniques and the currently used breathalyzer. The data were achieved from a 26-volunteer drinking study with people of different ages, biological sexes, and food habits. The sweat samples were obtained through pilocarpine electrophoresis similar to the Gibson and Cooke method [148], which allowed the sweat to be collected in gauze pads and analyzed. A minimum of 3 µL of sweat was required for this method.

These advances with biosensors in the field of forensics have produced a viable way for investigators to receive some information to pursue leads if DNA evidence is backlogged or not applicable using sweat and blood evidence found at the crime scene. Multiple enzymatic assays were developed for the differentiation of biological sex of an individual from fingerprint content, enabling an alternative or additional analysis for fingerprints depending on the clarity of the print for pictorial analysis. Blood was examined, and provides a viable methodology in order to show the time since deposition, TSD, and also an estimated age of the originator of the blood spot. Additionally, a field-deployable testing strip was developed for the determination of sweat, supplying clarity for a difficult-to-detect bodily fluid at crime scenes. Sweat was also tested in a laboratory setting for an alternative to breathalyzers for the detection and quantification of alcohol in sweat utilizing enzymatic assays and a colorimetric response. In the future, different drugs and illicit substances, such as THC, in addition to a more broad analysis of metabolites characteristic to certain habits or biological features can be examined for their use in forensics for providing a more deterministic and rapid analysis for forensic and law enforcement personnel in a sweeping suite of biosensor devices.

*Biometrics*

Currently, biometrics is another avenue of interest for biosensors [149]. This paper from Hair et al. uses the levels of three metabolites found in sweat in order to differentiate people. This analysis is performed using three enzymatic assays that each target the metabolites: Lactate, urea, and glutamate. The assay for lactate is the same one that was outlined in the sweat paper involving the paper strips. The assay for urea involves the enzymes urease and glutamate dehydrogenase. The final assay that is used for glutamate involves only glutamate dehydrogenase. These assays could be measured spectrophotometrically using either a redox dye in LOx/HRP or with conversions involving NADH and NAD^+^ in the other two. First, 50 mimicked sweat samples were run and compared, which showed that this method was viable as there was no overlap between the samples. Additionally, 25 authentic samples were analyzed, where the sweat was collected according to the same procedure as the Gibson and Cooke method mentioned above [148]. A multivariate analysis of variance statistic test (MANOVA), was performed for both the authentic and mimicked sample sets to determine if the combination of the three analytes were truly unique. Both sets produced *p*-values of <0.001 each. In addition, six ANOVA tests were performed between each analyte for the mimicked and authentic samples. All six of these tests also produced *p*-values of <0.001. These statistical values show that there is not only a significant statistical difference between individuals in the combination of the three analytes, but also a significant difference between each individual for a single analyte. The analysis shows that these three metabolites can be used in order to produce an individual’s “sweat profile,” enabling differentiation between individuals. The implications for this research are multidisciplinary as there are many forensic, cybersecurity, and point-of-care diagnostic applications that would benefit from this method.

This use of sweat content for biometric purposes is progressing but further research needs to be done for biometrics to be a reliable form of authentication using biosensor methodologies. The main future aspect that would need to be studied is a long-term study relating to the monitoring of the levels of the chosen metabolites in people and how the levels fluctuate over time relating to different factors such as stress, diet, and other day-to-day habits. For higher security when used for authentication, especially for higher-security systems and cybersecurity, additional metabolites would need to also be concurrently measured as well. This monitoring process would not only assist in the future of biometrics but also in existing disciplines such as clinical diagnostics.

*Cybersecurity*

Lastly, the use of biosensors for cybersecurity is a growing trend. The use of sensors for authentication of an individual and a novel methodology related to cryptography were recently developed [150,151]. The first paper represents a review with the aim of introducing a multi-assay wearable biosensor that would provide continuous tracking of a person’s sweat metabolites for authentication purposes. This review looks at many of the assays previously mentioned: ALT/LDH, ALT/POx/HRP, and GlDH, and some that were not mentioned: Alanine and glutamate assay with ALT, glutamate oxidase, and HRP; aspartate using the aspartate transaminase enzyme; and a combined version with all three of these new analytes in a single assay. By monitoring these assays, one can produce output data that would be beneficial in the authentication of a person with many cybersecurity applications. The second paper illustrates the use of three enzyme assays in order to encrypt a short message using a basic cipher [151]. The three enzymatic assays used involved HRP, lysozyme, and ALP. HRP and ALP were used as previously mentioned. Lysozyme breaks down cell walls from cells added during the assay to produce a reduction in signal at 450 nm, acting as the recognition element. The data resulting from the colorimetric assays are used in the encryption of a message. Provided that the receiver of the message performs the same experiment under the same conditions, the message will be properly decrypted. The data from these enzymatic assays act as “keys” that one use in order to lock and unlock data in relation to encryption.

This brief combination of cryptology and biosensors can have a large impact on the future of user authentication, cryptography, and unconventional computing as a whole. The processes outlined here can be combined with biometrics for user authentication, which is considered just as, or more, important compared to data security through encryption. In cryptography, the further research of other biosensor systems combined with stronger and more robust encryption methods can lead to the advancement of these systems to be used instead of random number generators, which have been controversial for use in cryptography since their inception [152]. Going even further, biosensors can be further researched for direct encryption of data to provide an alternative to the widely researched encryption via DNA [94,95,96,97,98,99,100,101,102,122,123,124,125,126,153,154,155,156,157].

## 3. Conclusions

Current research in biosensors has led to advancement in the use of biosensors on three fronts: Forensics, biometrics, and cybersecurity. In the field of forensics, the use of fingerprint material has been demonstrated to be capable of being used to determine the biological sex of a person through multiple methods. These methods relied on the detection of certain amino acids, some methods consisted of a broad detection of 23 amino acids, and some methods were much more selective and targeted down to a single amino acid. Additionally, the use of another medium, blood, allows one to deterministically estimate the amount of time that the blood has been outside the body for up to 2 days by analyzing the degradation of enzymes present in blood. Additionally, it was found that a single assay was able to not only estimate this time since deposition, but to also estimate the age of the originator. Sweat has been the third major medium in forensic endeavors, building upon the fingerprint analysis as fingerprints contain sweat. A novel methodology for a lactate-detecting on-site testing strip was developed in order to identify the presence of sweat due to the high concentration of lactate in sweat. This method was highly sensitive and required extremely small volumes of sweat to produce a tangible response. Sweat has also been examined for other purposes, showing that one can detect the presence of ethanol in sweat, providing another method of determining intoxication levels besides a breathalyzer or invasive blood techniques, in addition to detecting other drugs of abuse. Sweat was further analyzed for biometric purposes by comparing the levels of three metabolites found in sweat to differentiate individuals. Lastly, methods involving biosensors for both the authentication of individuals and for cryptography were developed, benefitting two major establishments of cybersecurity.

In the future, biosensors can further fulfill the expansion of these three fields with additional research. In the field of forensics, a wider array of metabolites may be examined for use in a device that would analyze a certain body fluid and provide more information relating to the habits and identifying information of the originator in addition to the biological sex and age mentioned previously. Additionally, more research can be performed in order to provide for a broad testing kit with higher accuracy and precision for various compounds, illicit and legal, for use in roadside testing to aid law enforcement officers. Biometrics and biosensors are closely related, as shown by the research seen here. Further analysis utilizing monitoring and other metabolite tracking will reinforce not only the strengths in the use of this methodology but also possibly reduce or remove the current unknowns and limitations for this method. Lastly, the use of different bioaffinity-based assay systems for cryptography for the use of different cipher systems will provide a reliable alternative to the random number generator systems used in cryptography today. This work in cybersecurity can also be combined with biometrics for user authentication for digital and evolving systems. Biosensors have been an important facet in the fields of clinical diagnostics, environmental processes, and military devices, and is a strong emerging technique in the fields of forensic science, biometrics, and cybersecurity. In these three fields, biosensors have produced considerable results thus far and have an auspicious future for further research.

## Figures and Tables

**Table 1:** Recent biosensor analysis techniques for sweat.

Protocol	Description	Technique	Analyte	LOD/Range	Ref.
Tattoo	Wearable skin tattoo for wireless signal transduction	Potentiometry	Sodium	0.1–100 mM	[71]
Tattoo	Wearable skin tattoo for pH monitoring	Potentiometry	pH	pH 3–7	[89]
Tattoo	Wearable skin tattoo for lactate monitoring	Potentiometry	Lactate	1–20 mM	[70]
Tattoo	Wearable skin tattoo for monitoring	Potentiometry	Ammonium	10^−4^–0.1 M	[90]
Superwettable bands	Multiplex method for on-body sampling	Colorimetric	pH	pH 4.5–7	[91]
			chloride	0–100 mM	
			glucose	0–15 mM	
			calcium	0–15 mM	
Screen-printed electrode	Monitoring of cystic fibrosis patients	Potentiometry	Chloride	2.7 × 10^−5^ mol/L	[92]
Janus textile bands	Multiplex method for on body sampling	Potentiometry	Glucose	18–40 µM	[93]
			Lactate	10 mM	
			Potassium	0.3–6.3 mM	
			Sodium	60 mM	
Wearable sensor	Stretchable, skin-attachable sweat sensor	Potentiometry, Carbon nanotubes, gold nanosheets	Glucose	10.89 µA mM^−1^ cm^−2^	[94]
			pH	71.44 mV pH^−1^	
Graphene electrochemical	Diabetes monitoring	Gold-doped graphene	Glucose	10 µM–0.7 mM	[95]
Microfluidic wearable sensor	Multiplex analysis for sensing in sweat	Colorimetric	Lactate	0–100 mM	[96]
			Chloride	0–mM	
			Creatine	0–1000 µM	
			pH	pH 5–8.5	
			Glucose	0–25 mM	
liquigel	Organic electrochemical transistor	Transistor	Lactate	0.3–1.3 mM	[97]
Direct iontophoresis	Sweat extraction and electrochemical analysis using smartphone	Potentiometry	Glucose	0–100 µM	[98]
			Chloride	20–80 mM	
Free amino acid analysis	Eccrine sweat amino acid composition	Cation chromatography and amino acid analyzer, GC-MS	Amino acids	-	[99]
Wearable Sensor	Chemical electrocardiogram and simultaneous metabolite monitoring	Amperometry	Lactate	0–28 mM	[100]
microfluidic	Sweat collection and analysis for kidney disorders	Colorimetric	Creatine	0–0.5 mM	[101]
			Urea	0–250 mM	
			pH	pH 5–7	
Wearable sensor	Integrated multiplex array for sweat analysis	Amperometry	Sodium	20–120 mM	[102]
			Potassium	2–16 mM	
			Glucose	0–200 µM	
			lactate	2–30 mM	
Wearable sensor	Monitoring for cystic fibrosis patients for sodium concentration	Potentiometry Atomic Absorption	Sodium	20–100 mM	[103]
Watch sensor	Monitoring of sodium levels	Potentiometry	Sodium	10^−4^ –10^−1^ M	[104]
Tattoo	Wearable skin tattoo for alcohol monitoring in sweat	Amperometry	Alcohol	0–36 mM	[74]
Wearable sensor	Drug monitoring via differential pulse	Voltammetry	Caffeine	0–40 x µM	[105]
Wearable sensor	Detection of THC and Alcohol	Voltammetry Amperometry	THC	0.5 µM	[76]
			Alcohol	0.1–1 mM	
